# Testing Strength of Biotic Resistance against an Introduced Fish: Inter-Specific Competition or Predation through Facultative Piscivory?

**DOI:** 10.1371/journal.pone.0031707

**Published:** 2012-02-20

**Authors:** J. Robert Britton

**Affiliations:** Centre for Conservation Ecology and Environmental Change, School of Applied Sciences, Bournemouth University, Fern Barrow, Poole, Dorset, United Kingdom; Argonne National Laboratory, United States of America

## Abstract

Biotic resistance is the process where aspects of the receiving environment inhibit the establishment and invasion of an introduced species. Resistance against an introduced fish can be through strong competition and/or predation from resident fishes. Here, the biotic resistance against introduced topmouth gudgeon *Pseudorasbora parva* (a highly invasive fish in Europe) by resident carp *Cyprinus carpio* was tested in experimental mesocosms. The introduction scenario was six adult *P. parva* (three male, three female) on a single occasion. Resistance to their establishment was provided by three and six resident *C. carpio* whose effects on *P. parva* growth and reproduction were compared to a Control (no resident fish at the time of introduction) and treatments containing three and six *P. parva*. After 120 days, the growth rates of the introduced *P. parva* were significantly depressed in *C. carpio* presence and in mesocosms with three *C. carpio* present, significantly decreased numbers of 0+*P. parva* were recorded. Where six *C. carpio* were present, no 0+*P. parva* were recorded, indicating resistance strength increased with carp abundance. In contrast, there were no differences in *P. parva* reproduction and growth rates between the Control and treatments containing conspecifics. Stable isotope analysis (δ^15^N, δ^13^C) revealed *C. carpio* were feeding at one trophic level above 0+*P. parva*, suggesting the process of resistance was predation (facultative piscivory) rather than competition. Thus, if *P. parva* are to establish and invade following an introduction, they must overcome this biotic resistance from cyprinid fishes such as *C. carpio*.

## Introduction

The probability of an introduced species surviving, establishing a sustainable population and then developing invasive populations is dependent on the interaction of numerous factors in the receiving environment [1]. Survival and establishment of the introduced species may be inhibited by environmental factors, such as climate and habitat [2]. If environmental conditions are suitable then biotic resistance can be important through inhibiting establishment processes [3], [4], [5]. This resistance may result from the diversity of the communities in the receiving environment [6], [7], [8] or from the presence of predators or strong competitors that impede survival and reproduction of the introduced propagules [1], [4], [9], [10]. In aquatic environments, resistance against invasions have been shown for groups of species including introduced crabs [1], [2], zooplankton [11] and clams [12]. Examples of biotic resistance against introduced fish are relatively rare. Fish assemblages in Californian streams that have not been subjected to human disturbance were able to resist invasions from introduced fish through biotic factors including predation [4]. Establishment of introduced non-indigenous brook trout *Salvelinus fontinalis* in Idaho, USA, was not, however, resisted by the native rainbow trout *Oncorhyncus mykiss*, with habitat characteristics being more important in determining invasion success [13].

The topmouth gudgeon *Pseudorasbora parva* is a small fish of the Cyprinidae family that is native to East Asia that was introduced into Europe in the 1960s and has since proved highly invasive [14], [15]. It is now present in at least 32 countries and is continuing to disperse [14]. Consequences of invasions for native fishes include increased inter-specific competition for resources [16] and disease transmission [17], [18]. In the UK, invaded lakes tend to be characterised by highly abundant populations, with densities often above 60 m^−2^ [16], [19]. The establishment of such large *P. parva* populations within fish communities that are often composed of several native and resident fishes [16] suggests biotic resistance against their establishment is minimal. What is unknown, however, is the proportion of introductions that have not resulted in establishment and the processes by which introduction failure may occur. This is, in part, due to detection of introduced *P. parva* only tending to occur after establishment of a relatively large population due to issues of imperfect detection at low population sizes [20]. Consequently, the established and invasive *P. parva* populations in the UK (and elsewhere in Europe) may represent the outcomes of only a small proportion of all *P. parva* introductions.

The aim of this study was to investigate whether establishment of *P. parva* could be inhibited by a resident fish in the receiving water via competition and/or predation processes. Rather than use uncontrolled wild populations for the study, where information on introduction characteristics, such as propagule pressure (number of introduced individuals) [21], are unknown, an experimental approach was adopted using small replicated mesocosms. This enabled the same number of propagules to be released across a range of introduction scenarios. The resisting species was the common carp *Cyprinus carpio*. Although also an invasive fish in many parts of the world, it is now encountered regularly throughout European freshwaters [22]. In the UK, regulatory authorities tend to treat it in the same manner as native fishes, with approximately 6 million carp legally stocked into lake fisheries between 1998 and 2008 [22]. Consequently, the species is present in virtually all of ponds and lakes in the UK where *P. parva* have been introduced [16], [19] and thus are a representative species for testing biotic resistance against *P. parva* establishment. Moreover, both species are originally native to East Asia and so *C. carpio* may also be potentially considered as a natural competitor, and given their omnivorous foraging that can include facultative piscivory [23], perhaps even a natural predator. The objectives were to (i) identify whether *C. carpio* is able to resist *P. parva* establishment; (ii) determine the role of *C. carpio* abundance in determining the strength of biotic resistance; and (iii) where resistance is shown against *P. parva*, determine the role of competition and predation in this. It was predicted that increased *C. carpio* abundance would result in reduced establishment rates of *P. parva*, with resistance mediated through inter-specific competition for food resources.

## Materials and Methods

### Ethics statement

All animal work was conducted in accordance to national and international guidelines to minimize discomfort to animals. All regulated procedures completed under the Animals (Scientific Procedures) Act 1986 were licensed by the UK Home Office under project licence number PPL 30/2626. The Ethics Review Panel of the School of Applied Sciences of Bournemouth University approved this project licence.

### Experimental design

The experimental mesoscosms were 1×1 m in diameter, 1.25 m in depth, were positioned adjacent to each other and were located outdoors in Southern England; a total of 20 were used. The surface of each was covered with 10 mm nylon mesh to prevent entry of piscivorous animals. A substrate of gravel was provided, along with a smaller number of larger stones to provide reproductive *P. parva* males with territorial areas and nesting sites. The mesocosms were used to design five experimental treatments (including a Control) that were replicated four times (Table 1). Sex ratios of the *P. parva* were 1 male: 1 female, as this ratio is typically encountered in the wild [14]. The initial introductions of fish into the mesoscoms were in May 2011 and the *P. parva* were introduced at the beginning of June 2011 (Table 1). The rationale of the experimental design (Table 1) was: (i) the Control would provide no resistance to *P. parva* establishment as there were no other fish present; (ii) the presence of three and six *C. carpio* in the two treatments would potentially provide resistance to invasion in a hierarchical manner; and (iii) the presence of three and six *P. parva* in the final two treatments would provide comparison of the effect on establishment of the same number of conspecifics as the number of *C. carpio* in their treatments. Prior to the introduction of the *P. parva* in June 2011, the fish were taken to the laboratory where they were anaesthetised with MS-222 and fin clips taken (pelvic fins). In addition to helping provide the tissue samples for stable isotope analysis (see next section), this provided a method of non-intrusive, individual marking of the fish according to sex (left pelvic, right pelvic, no clip for the three male and three female *P. parva*) and so tags were not necessary. Note *P. parva* can be differentiated by sex according to body colour and morphology [14]. The fish were also measured (fork length, nearest mm) and then following their recovery, they were transported to the mesocosms and released.

**Table 1:** Overview of the Control and Treatments used in the biotic resistance experiment.

Treatment	Starting number of fish (May 2011)	Introduced fish (June 2011)*
Control	0	6 *P. parva*
Treatment 1	3 *C. carpio*	6 *P. parva*
Treatment 2	6 *C. carpio*	6 *P. parva*
Treatment 3	3 *P. parva*	6 *P. parva*
Treatment 4	6 *P. parva*	6 *P. parva*

The *Cyprinus carpio* and *Pseudorasbora parva* were all 65 to 80 mm (fork length). Each treatment was replicated four times.

*At a sex ratio of 1M: 1F.

The mesocosms were then left for 120 days. The only disturbance in this period was sampling of water chemistry, with parameters recorded including dissolved oxygen and ammonia. No significant differences in any chemical parameter were detected across the experimental period and so were not considered further (ANOVA, *P*>0.05). At the conclusion of the experimental period, the treatments with three and six *C. carpio* (Table 1) had samples of phytoplankton, epilithic algae (from the stones originally placed on the benthos) and leaf litter (small leaves entering the mesoscosms through the mesh covers) taken prior to the water being partially drained from all mesocosms. The fish community of each mesocosm was then able to recovered; data recorded were the numbers of the original *P. parva* (hereafter referred to as adult *P. parva*) and *C. carpio*, and the number of young-of-the-year *P. parva* (hereafter referred to as 0+*P. parva*). In all mesocosms, the *C. carpio* and adult *P. parva* were all recovered. These fish were then taken to the laboratory where they were euthanized with an overdose of anaesthetic (MS-222), tissue samples (fin clips) taken for stable isotope analysis (taking tissue from the re-grown areas where an original fin clip was taken), and the lengths recorded for all *P. parva*.

### Data analysis

Establishment success was defined in the experiment as the *P. parva* reproducing and having 0+fish present at the end of the experimental period (i.e. successful reproduction and production of young-of-the-year that would subsequently recruit into the mature stock). The effects of inter-specific competition was assessed through the growth rate of the adult *P. parva* using incremental lengths (*IL*; mm d^−1^), calculated by [*L*_t+1_−*L*_t_]/t, where *L*_t_ and *L*_t+1_ was the starting length and *L*_t+1_ the final length of the fish, *t* was the duration of the experimental period (120 days). To identify the trophic relationships between the species and their putative food resources, stable isotope analysis was completed for the mesocosms used in the *C. carpio* treatments (Table 1). This provided values of δ^15^N (indicator of trophic level) and δ^13^C (indicator of energy source) [24] to reveal the trophic relationships between the *C. carpio* and *P. parva* and their putative food resources. Trophic positions (TP) for each individual fish were calculated using the formula: TP = [(fish δ^15^N−mean putative food source δ^15^N)/3.4]+2, where 3.4 represents a widely used single trophic level fractionation in δ^15^N, and 2 corresponds to the trophic level of primary consumers [25], [26]. All samples were dried for 24 hours at 60°C before being processed at the Cornell Isotope Laboratory, Cornell University, New York, USA.

### Statistical analyses

Data to determine if differences in the lengths, incremental lengths, stable isotope values of δ^15^N and δ^13^C and trophic positions were significant between the species were initially tested for normality and log transformed where necessary. Parametric tests were then used to test for significant differences in mean values using ANOVA; ANCOVA (in General Linear models, GLM) was used where covariates had to be controlled in the analyses, such as the allometric effect of fish length. The ANCOVA models were only considered valid and used subsequently when the assumptions were met that variances were equal between the groups (Levene's test, *P*>0.05), there was no interaction between the covariates and the groups (homogeneity of the regression slope; *P*>0.05) and where the test results were significant, post-hoc power analysis indicated statistical power >0.80. Although mixing models were also used to test the stable isotope data in relation to determining the relative contributions of the putative food resources to fish diet, their outputs were considered unreliable due to issues with the high standard deviations that resulted from variance in the values of the putative food resources. In all cases, where error is provided around mean values, they represent 95% confidence limits unless stated otherwise. All statistical tests were completed in SPSS v. 16.0 and assessed at α = 0.05.

## Results

**Figure 1:**
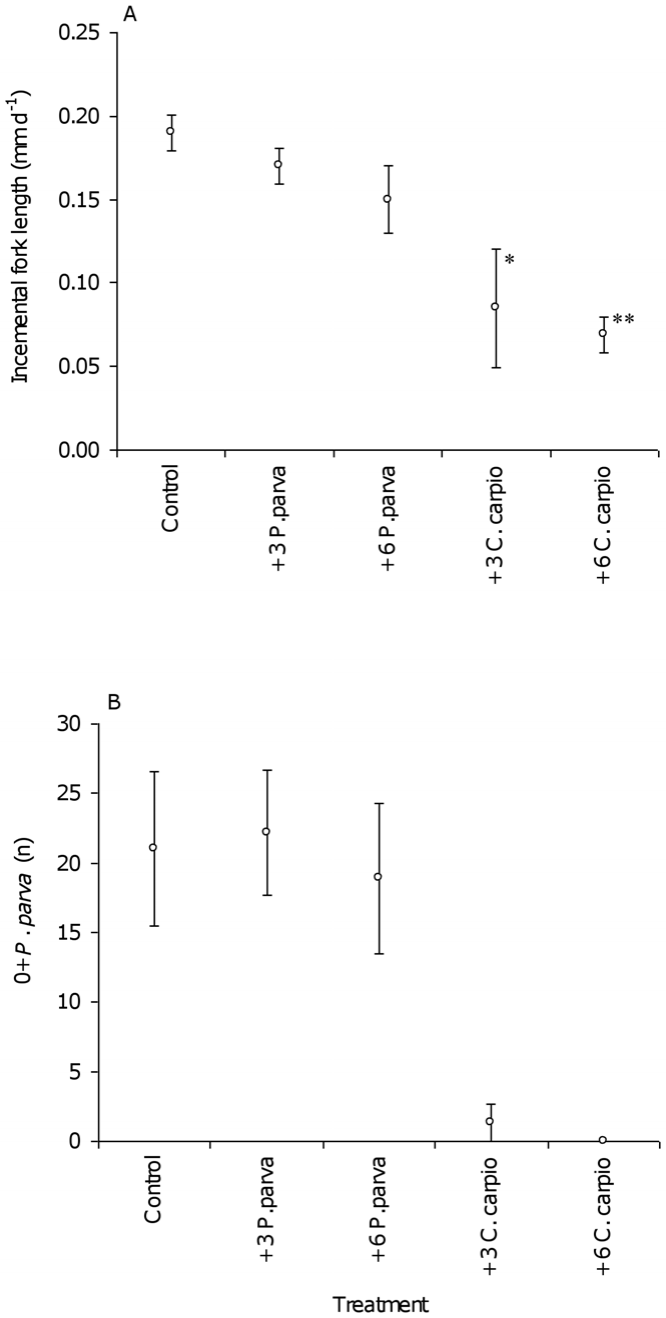
*Pseudorasbora parva* growth rates and reproduction in the experimental control and treatments. (A) growth, as incremental fork length and where *P<0.05; **P<0.01 compared to the control; and (B) reproduction, as the number of 0+fish produced.

The growth rates of the introduced *P. parva* were significantly depressed in the *C. carpio* treatments when compared to the Control (Fig. 1a; Table 2). By contrast, the growth rates of the original *P. parva* in the presence and three and six conspecifics were not significantly different (Table 2; Fig. 1a). In all cases, the growth rates of the adult *P. parva* were independent of their starting lengths (R^2^ = 0.08; F_1,128_ = 0.87, P>0.05).

**Table 2:** Effects of the experimental treatments and associated co-variates on the incremental lengths of *Pseudorasbora parva*.

Effect	Incremental length
Sex	F_1,13_ = 0.46, *P*>0.05
Starting length	F_1,13_ = 0.06, *P*>0.05
Experimental treatments	F_4,10_ = 6.43, *P*<0.05

Difference between treatments (mean ± S.E.)		
Control	+3 *P. parva*	0.03±0.05
	+6 *P. parva*	0.05±0.05
	+3 *C. carpio*	0.11±0.04*
	+6 *C. carpio*	0.13±0.02**

Sex and starting length were the covariates in the ANCOVA model; corresponding differences, indicated by pairwise comparisons with Bonferroni adjustments for multiple comparisons, between the Control and the other Treatments are displayed.

*P<0.05;

**P<0.01.

The reproductive success of *P. parva*, expressed as the number of 0+fish present per treatment at the conclusion of the experimental period, was similar between the Control and the *P. parva* treatments (Fig. 1b). Between 15 and 28 0+*P. parva* were recovered from these mesoscoms, with no significant differences between the Control and Treatments (Mann Whitney: Control vs. three conspecifics Z = 0.56, *P*>0.05; Control vs. six conspecifics Z = 0.87, *P*>0.05). The mean length of the 0+fish across these treatments was 22.9±2.1 mm, with differences not significant between treatments (F_2,281_ = 1.24, *P*>0.05). By contrast, there were no 0+*P. parva* recovered from the mesoscosms where six *C. carpio* were present (Fig. 1b). In the treatment with three *C. carpio*, three of the four replicates had 0+*P. parva* present, although numbers were only between one and four fish (Fig. 1b), significantly lower than the Control (Z = 7.84, *P*<0.01).

**Figure 2:**
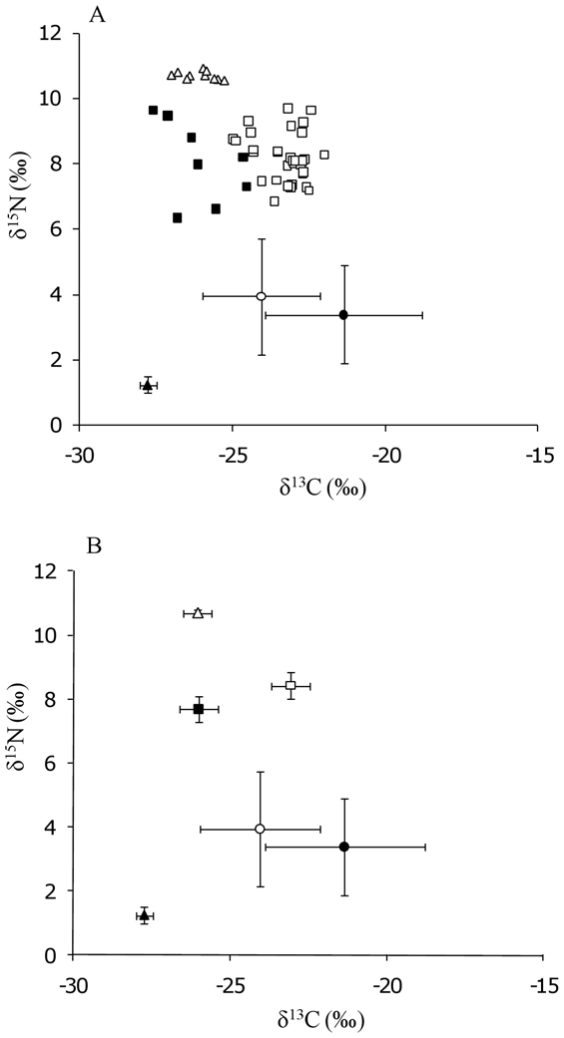
Stable isotope biplots of *Cyprinus carpio* (Δ), *Pseudorasbora parva* adults (□) and 0+*P. parva* (▪) and the putative food and basal resources (○ phytoplankton; • epilithic algae; ▴ leaf litter). A) Individual values of δ^13^C and δ^15^N for the fishes are displayed; B) all values of δ^13^C and δ^15^N are means, where variance around the mean are 95% confidence limits.

Stable isotope analysis was completed for the treatment where three *C. carpio* were present, as these were the only mesocosms where *C. carpio*, adult *P. parva* and 0+*P. parva* were all present together. Across the four replicates, values of δ^15^N and δ^13^C were not significantly different for each species and grouping (δ^15^N: *C. carpio* F_1,15_ = 1.12, *P*>0.05; adult *P. parva* F_1,22_ = 1.68, *P*>0.05; 0+*P. parva* F_1,6_ = 0.78, P>0.05; δ^13^C: *C. carpio* F_1,15_ = 0.98, *P*>0.05; adult *P. parva* F_1,22_ = 1.47, *P*>0.05; 0+*P. parva* F_1,6_ = 0.98, P>0.05). Consequently, the isotope data were combined across these mesocosms. The GLMs and stable isotope biplot revealed the *C. carpio* were feeding at a higher trophic level than both groups of *P. parva* (Fig. 2a,b; Table 3), with the mean trophic position of the 0+*P. parva* being 3.22±0.13, adult *P. parva* 3.43±0.11 and *C. carpio* was 4.08±0.03. The overall differences in the TP values were significant according to the species' groups (ANOVA: F_2,40_ = 22.10, *P*<0.01). Tukey's post-hoc tests revealed the significant differences were between *C. carpio* and 0+*P. parva* (0.86±0.13, P<0.01) and *C. carpio* and adult *P. parva* (0.65±0.11, *P*<0.01), but not between the two groups of *P. parva* (0.21±0.10, *P*>0.05).

**Table 3:** Outputs of the general linear models testing differences in trophic niche between the species.

(a) δ^15^N	
Effect	Mean δ^15^N
Species	F_2,33_ = 4.48, *P* = 0.02
Fish length	F_2,33_ = 0.78, *P*>0.05
Species×fish length	F_2,33_ = 1.11, *P*>0.05

Difference between species (mean ± S.E.)		
*C. carpio*	Adult *P. parva*	2.21±0.55‰, *P*<0.01
*C. carpio*	YoY *P. parva*	2.55±0.81‰, *P*<0.01
Adult *P. parva*	YoY *P. parva*	0.36±0.42‰, *P*>0.05

(b) δ^13^C	
Effect	Mean δ^15^N
Species	F_2,33_ = 5.96, *P*<0.01
Fish length	F_2,33_ = 2.99, *P*>0.05
Species×fish length	F_2,33_ = 2.98, *P*>0.05

Difference between species (mean ± S.E.)		
*C. carpio*	Adult *P. parva*	3.84±1.51‰, *P*<0.05
*C. carpio*	YoY *P. parva*	1.59±1.00‰, *P*>0.05
Adult *P. parva*	YoY *P. parva*	2.26±0.76‰, *P*<0.02

(a) Differences between species for δ^15^N; (b) differences between species for δ^13^C. Species were defined as Cyprinus carpio, mature *Pseudorasobora parva* and 0+(YoY) *Pseudorasobora parva*. Fish length was the covariate.

## Discussion

Under a scenario of a single *P. parva* introduction event and a set number of released propagules (×6) at an equal sex ratio, reproduction and survival of 0+*P. parva* was apparent in the Control and treatments containing conspecifics, suggesting establishment was occurring. By contrast, the presence of three *C. carpio* in the treatments suppressed 0+*P. parva* survival and where six *C. carpio* were present, no 0+fish were found at the end of the experimental period. Thus, this strongly suggests the *C. carpio* were successfully resisting the establishment of the introduced *P. parva*, with the resistance strength increasing with carp number, as per the prediction. The outcome of the stable isotope analysis strongly suggested that the mechanism of this biotic resistance was predation via facultative piscivory rather than inter-specific competition, given that *C. carpio* were feeding at approximately one trophic level above 0+*P. parva*. Predation pressure increased as *C. carpio* numbers increased, whereby no 0+fish were present at the end of the experimental period in mesocosms with six carp present. Moreover, the carp were not predating the adult *P. parva* as these were all recovered at the conclusion of the experiment. In wild studies, stable isotope ecology of sympatric *C. carpio* and *P. parva* has suggested overlaps in trophic niche rather than segregation, with sharing of common food resources across the entire length ranges of both species [16]. However, in that particular study, predation on *P. parva* by other species of the Cyprinidae family was suggested, although this was insufficient to prevent formation of a large *P. parva* population [16]. In other studies investigating the ecological consequences of introduced fishes using stable isotope analysis, the effects of predation tends to be from the invading species, with deleterious impacts recorded on native fishes from, for example, introductions of small mouth bass *Micropterus dolomieu* and rock bass *Ambloplites rupestris* [27] and European catfish *Silurus glanis* [28]. In other isotopic studies involving *C. carpio*, outputs suggest they are rarely piscivorous [29], although elevated levels of δ^15^N were recorded for carp in Lake Naivasha, Kenya, where stomach contents analysis also revealed the presence of both fish and invasive crayfish in their diet [23].

Whilst the growth rates of the adult *P. parva* were depressed in the presence of *C. carpio*, suggesting competitive interactions were also a potential mechanism in the biotic resistance, the trophic outputs suggested these fishes were utilizing separate food resources (basal resources for *P. parva*, 0+*P. parva* for *C. carpio*). Whilst there tends to be growth consequences for both species when used experimentally in confined spaces (such as aquaria) with additional biomass as important as additional density in determining the extent of the depressed growth [30], this did not appear to be the resistance mechanism here due to the absence of sharing of trophic space. Instead, the depressed growth of these adult *P. parva* may instead have been indirectly inhibited by *C. carpio*; as their 0+fish continued to be predated, the introduced adults may have continued to expend their energies on reproduction (e.g. maturation of gonads, continued expression of spawning behaviours, nest building etc) rather than somatic growth. This, however, must remain speculative in the absence of firmer evidence.

The outputs of this study strongly suggested that *C. carpio* populations can resist introduced *P. parva* from establishing sustainable populations. Given these are experimental data, however, then it is important to note that many experimental studies that deal with ecological interactions such as foraging and competition suffer from a range of inherent issues [31]. For example, it can be rare for experimental data to match field observations, as the spatial constraints used experimentally can cause unnaturally intense interactions that result in an over-extrapolation of laboratory data [32]. In this study, the mesocosms were relatively small and although relatively low fish numbers were being used, there was little opportunity for the fishes to segregate in their resource use. Indeed, in larger systems, should some 0+*P. parva* survive and facilitate their establishment, then given their ability to subsequently form highly abundant populations [16], and forage on the eggs of other fishes [14], this means subsequent detrimental impacts may develop on the reproduction and recruitment of species such as *C. carpio*. Thus, should resistance by *C. carpio* fail to prevent *P. parva* establishment, then resilience against detrimental impacts may be limited [16]. In addition, the conditions provided in the mesocosms were unlikely to have represented more complex natural situations [33] where it was likely that there would have been additional fish species present in the community that were nested within a more complex food web, along with the presence of a more heterogeneous habitat; both would have influenced the outcome of the interactions of the fishes. Moreover, in the wild, the fishes would have also been subject to stochastic events arising from inclement weather (e.g. periods of freezing winter conditions of differing severity and duration) that could not be considered here. Indeed, the characteristics of the winter period may play an important regulatory role in the reproductive traits of *P. parva* [14] and so may influence the outcomes of their interactions with species such as *C. carpio*.

Consequently, due caution must be given to any inferences made from the experiments conducted here for scaling up to the more complex systems, and also in situations where propagule pressure from *P. parva* was higher than in the experiment, as this may increase establishment probability [21]. Nevertheless, the use of such controlled and replicated conditions in this experiment was capable of demonstrating the mechanism of resistance that would have to be overcome by *P. parva* when introduced into waters in the UK (and beyond) where cyprinid fishes, such as *C. carpio* are already present within the resident fish community.
